# Which is the best treatment on a 2 cm complete endophitic tumor on the posterior side of the left kidney?

**DOI:** 10.1590/S1677-5538.IBJU.2016.01.04

**Published:** 2016

**Authors:** Juan Arriaga, Rene Sotelo

**Affiliations:** 1 University of Sonora, Hospital CIMA, Hermosillo Sonora, México;; 2 USC Institute od Urology, University Southern California, USA

**Keywords:** Therapeutics, Neoplasms, Cryosurgery, Nephrectomy

## Opinion: Robotic partial nephrectomy

The routine use of abdominal imaging has led to increased detection of small renal masses incidentally, even before they cause symptoms (1). Partial nephrectomy is now the standard therapy for the treatment of small renal masses in stage T1a and even for certain patients with T1b tumors, offering equivalent cancer control to radical nephrectomy with better preservation of renal function and improving survival (2). The recurrence-free survival at 5 years for small renal masses less than 4 cm and 4-7 cm is about 96% and 83% respectively (3). It has been found that radical nephrectomy can lead to an increased risk of chronic kidney disease (4), and is associated with a higher risk of adverse cardiac events, hospitalization and death (5).

The location of the tumor and the endophytic component can be the determining factors on the feasibility and the degree of difficulty in performing a partial nephrectomy, even more important to consider than just the size of the tumor.

A completely intraparenchymal tumor is defined as an injury that is completely surrounded by normal renal parenchyma on all sides (6) or one located at a distance less than 5 mm of the collecting system or hilar vessels without exophytic component (7).

Partial nephrectomy is the treatment of choice for a tumor 2 cm diameter (3, 8) completely intrarenal on the posterior side of the left kidney, considering the context of clinical presentation such as age and comorbidities, even when the contralateral kidney is healthy.

We must consider that partial nephrectomy itself requires many surgical skills, but for a completely intrarenal tumor, partial nephrectomy is very challenging due to difficulties in the exact location of the tumor, complete resection with negative margins but not exceeding the resection of healthy tissue as well as obtaining the perfect hemostasis and renal reconstruction sutured safe, besides the risk of injury to underlying structures of the renal pelvis (9). Intraoperative ultrasonography is absolutely essential especially when tumor is completely intrarenal as it enables optimal placement of the incision (10). Robotic optical system allows three-dimensional vision of deep renal tissue for better identification of pathological tissue and articulated robotic rotary instruments allows proper closure and suture cavity surrounding healthy tissue. The robotic transabdominal approach offers more working space and greater anatomical references and even when the lesion is located on the posterior side, full kidney can be mobilized to achieve optimal exposure (11) with equivalents results than the open technique (11-13). The robotic system technologies have evolved so fast that the new systems already allow overlapping surgery ultrasonographic images in real time (TilePro^TM^, da Vinci® *Si* HD, Intuitive Surgical Inc., Sunnyvale, CA) for the best performance in this kind of procedures improving expectations of intraoperative and postoperative results.

The complete removal of the tumor may be performed by a circumferential incision of the affected renal segment, achieving partial nephrectomy that includes all the tumor. In cases where the tumor has a central location and nonpolar, it can be considered a linear vertical incision along the rear face of the kidney, such as that in the anatrophic nephrotomy, in order of major greater exposure of the tumor and more healthy tissue preservation, which itself would be a tumorectomy rather than a partial nephrectomy.

Anatrophic nephrotomy was described for the first time (14) for staghorn calculi. They used as a reference an imaginary avascular line located between renal portions irrigated by anterior and posterior segmentary branches of the main renal artery, known as Brodel line and thus has been probed that is less like to injure the intrarenal blood vessels of larger caliber compared to other renal surgery incisions, moreover no other approach offers three-dimensional exposure of deep intrarenal tissue.

This surgical approach has been historically used to treat great complex kidney stones and not for renal tumors. The proven benefits of this approach led us to consider addressing the anatrophic nephrotomy for optimal exposure and carry out the removal of a small tumor completely intrarenal (15, 16) as here, seeking maximum preservation of normal renal tissue.

## CONCLUSIONS

Robotic partial nephrectomy to a completely intrarenal tumor is feasible, safe and effective in experienced hands. However, it is an advanced technique that should only be performed by surgeons with extensive experience in robotic renal surgery. Intraoperative ultrasound is mandatory to guide tumor resection successfully. Regarding tumorectomy by anatrophic incision, may be an option for special cases and in expert hands, prospective studies are needed with larger numbers of patients to evaluate the oncological and functional results and its potential complications of this technique.
